# Supplementary material for: Prolonged persistence of mutagenic DNA lesions in somatic cells
Source: Nature. 2025 Jan 15;638(8051):729–38. doi: 10.1038/s41586-024-08423-8 (PMC11839459; doi:10.1038/s41586-024-08423-8)
Supplement: Supplementary file 1 — Supplementary Fig. 1. Bootstrap support for the phylogeny nodes defining the PVVs. Supplementary Fig. 2. PVVs for which either the lesion node, or lesion repair node are different in the IQ-tree phylogeny. Supplementary Fig. 3. PVVs for which either the lesion node, or lesion repair node has low bootstrap support [file 41586_2024_8423_MOESM1_ESM.pdf]

nature portfolio

https://doi.org/10.1038/s41586-024-08423-8

Supplementary information

Prolonged persistence of mutagenic DNA lesions in somatic cells

In the format provided by the authors and unedited

Supplementary Information

Prolonged persistence of mutagenic DNA lesions in somatic cells

Authors

Michael Spencer Chapman[1,2], Emily Mitchell[1,3,4], Kenichi Yoshida[1], Nicholas Williams[1], Margarete A. Fabre[1,3,4], Anna Maria Ranzoni[1], Philip S. Robinson[1], Lori D Kregar[1], Matthias Wilk[5], Steffen Boettcher[5], Krishnaa Mahbubani[6,7], Kourosh Saeb Parsy[6,7], Kate H. C. Gowers[8], Sam M. Janes[8], Stanley W. K. Ng[1], Matt Hoare[9], Anthony R Green[3,4], George S. Vassiliou[1,3,4], Ana Cvejic[1,3,4,10], Markus G. Manz[5], Elisa Laurenti[3,4], Iñigo Martincorena[1], Michael R Stratton[1], Jyoti Nangalia[1,3,4], Tim H. H. Coorens[1,11], Peter J. Campbell[1,3,4]

Supplementary Figure 1. Bootstrap support for the phylogeny nodes defining the phylogeny-violating variants.

Supplementary Figure 2. Phylogeny-violating variants for which either the lesion node, or lesion repair node are different in the IQ-tree phylogeny.

Supplementary Figure 3. Phylogeny-violating variants for which either the lesion node, or lesion repair node has low bootstrap support.

Supplementary Table 1. Characteristics of subjects

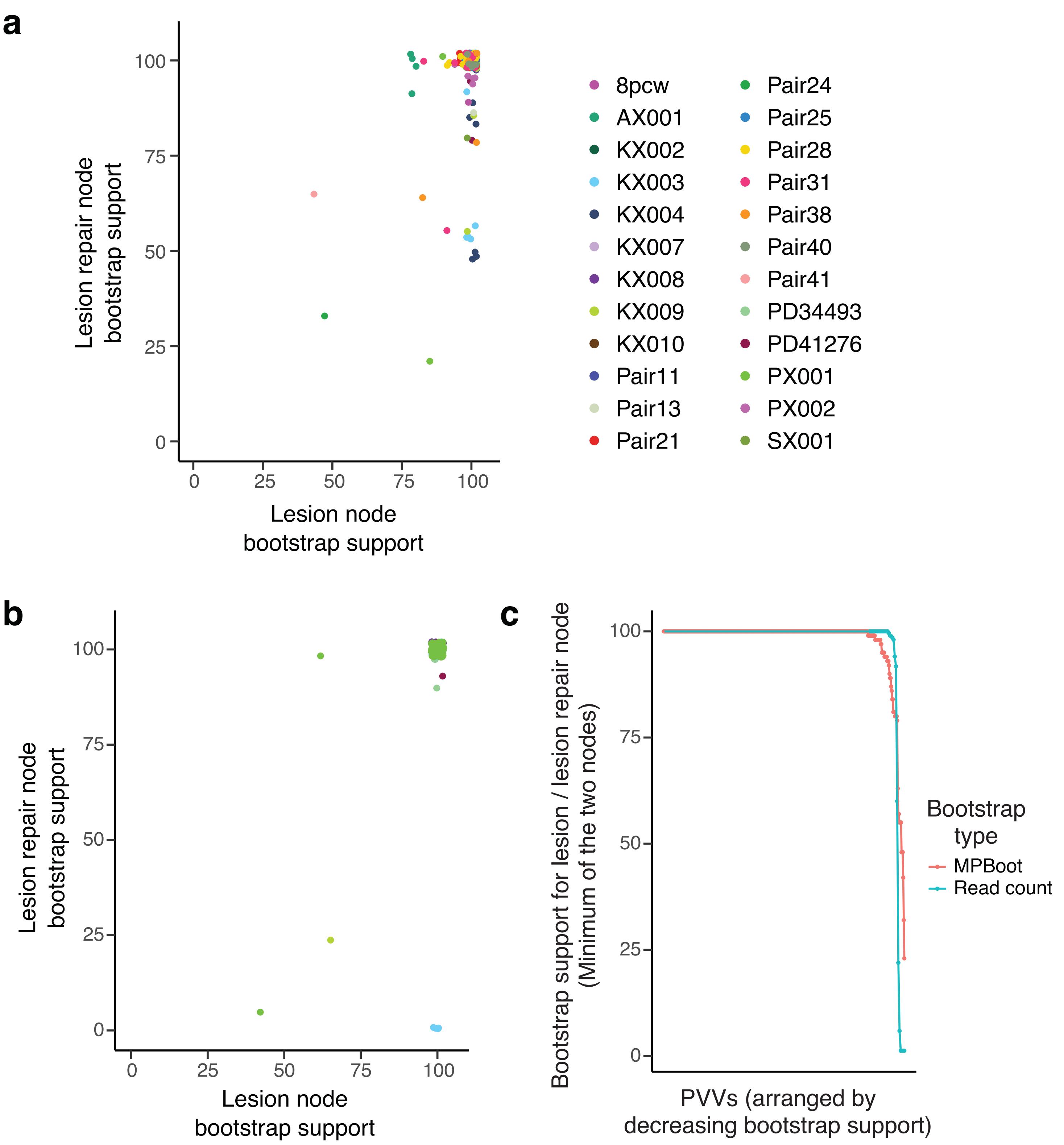

Supplementary Figure 1. Bootstrap support for the phylogeny nodes defining the phylogeny-violating variants. a, Jittered scatter plot showing the MPBoot bootstrap support values of the 'lesion node' and 'lesion repair node' of each of 440 PVVs. 90% of points are concentrated in the dense area in the top right where both nodes have >98% bootstrap support. b, as in a, but for the read count bootstrap support, and for a subset of 202 PVVs. c, Dot plot showing the MPBoot and readcount bootstrap support for all assessed PVV lesion/ lesion repair nodes (the minimum support of the two) arranged by decreasing support values. Points are joined by a line to better illustrate the trend. PVV, phylogeny-violating variants.

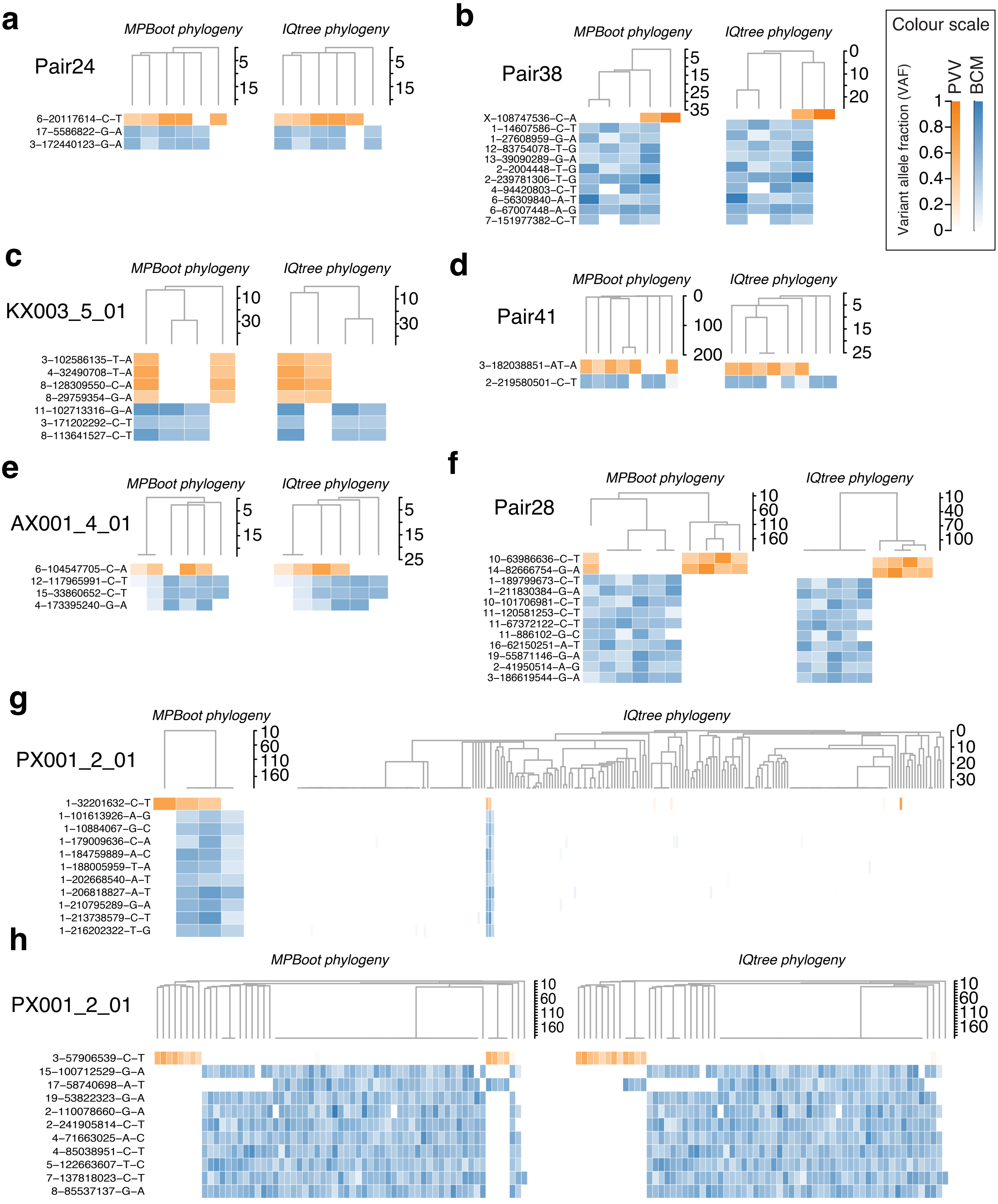

Supplementary Figure 2. Phylogeny-violating variants for which either the lesion node, or lesion repair node are different in the IQ-tree phylogeny. a-h, The clade defined by the PVV lesion node has been extracted from the full MPBoot phylogeny, shown on the left. On the right is the relevant clade extracted from the IQ-tree phylogeny, chosen as the latest clade containing all samples found within the MPBoot lesion node clade. These clades are truncated to better illustrate the features near the root. The y axis is molecular time, stated relative to the time of the lesion node (i.e. the root of the subtree shown). The heatmaps show the VAFs of mutations on a scale of white (absent) to orange (for the PVVs) or blue (for the branch-creating mutations, limited to ≤10 for visualisation). Mutation references are shown in the format Chromosome-Position-Reference base-Mutant base. PVV, Phylogeny-violating variant; BCM, Branch-creating mutations.

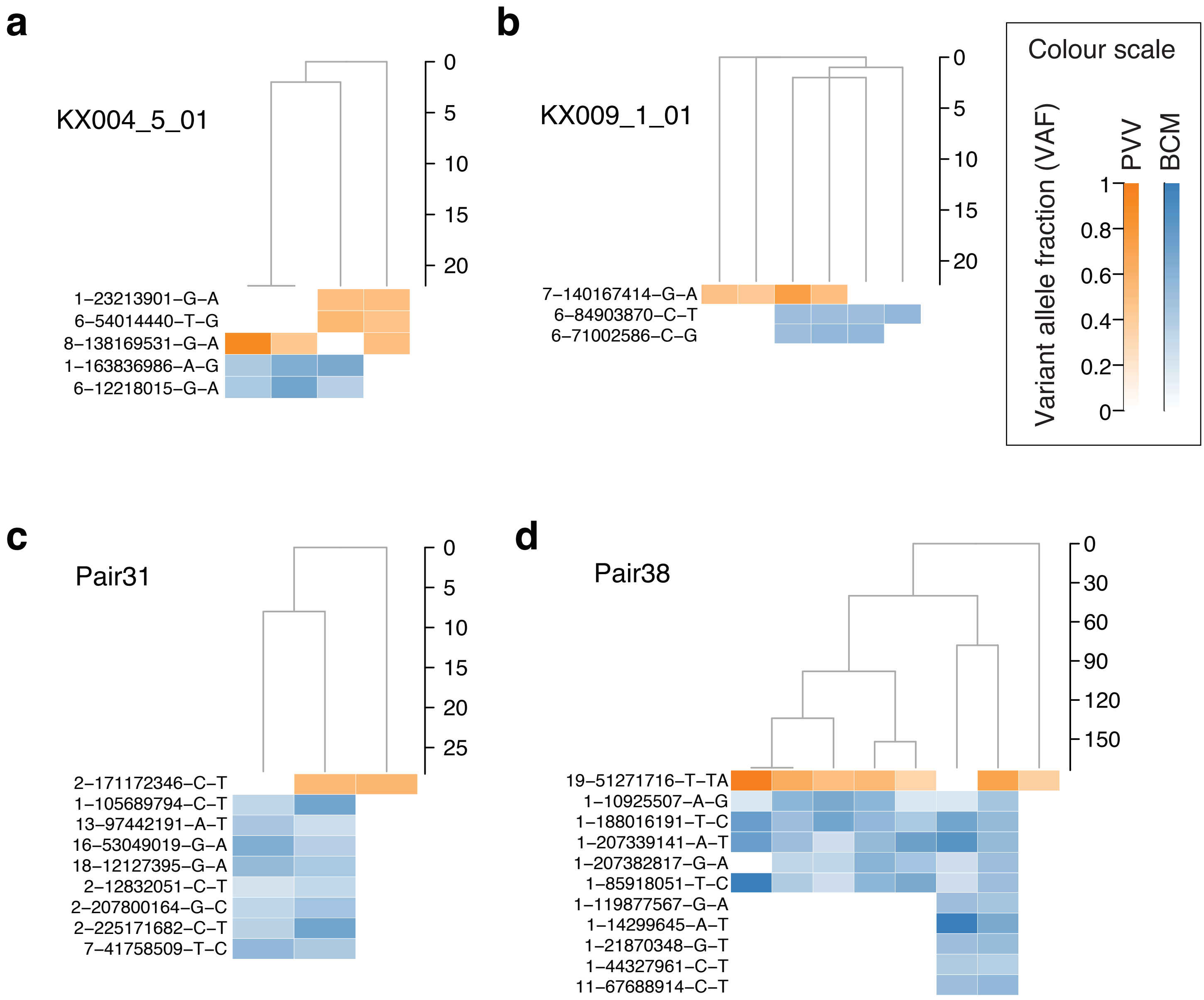

Supplementary Figure 3. Phylogeny-violating variants for which either the lesion node, or lesion repair node has low bootstrap support. This figure includes variants where either the lesion or lesion repair node have bootstrap support values <80% by either the MPBoot or read count bootstrap assessments. However, it does not include those already shown in Rebuttal Fig. X. a-d, the clade defined by the PVV lesion node has been extracted from the full MPBoot phylogeny and truncated to better illustrate the features near the root. The y axis is molecular time, stated relative to the time of the lesion node. The heatmaps show the VAFs of mutations on a scale of white (absent) to orange (for the PVVs) or blue (for the branch-creating mutations, limited to ≤10 for visualisation). Mutation references are shown in the format Chromosome-Position-Reference base-Mutant base. PVV, Phylogeny-violating variant; BCM, Branch-creating mutations.
