# Supplementary material for: Prolonged persistence of mutagenic DNA lesions in somatic cells
Source: Nature. 2025 Jan 15;638(8051):729–38. doi: 10.1038/s41586-024-08423-8 (PMC11839459; doi:10.1038/s41586-024-08423-8)
Supplement: Supplementary file 2 — Reporting Summary [file 41586_2024_8423_MOESM2_ESM.pdf]

nature portfolio

Corresponding author(s): Peter Campbell
Last updated by author(s): 1st August, 2024

Reporting Summary

Nature Portfolio wishes to improve the reproducibility of the work that we publish. This form provides structure for consistency and transparency in reporting. For further information on Nature Portfolio policies, see our Editorial Policies and the Editorial Policy Checklist.

Statistics

For all statistical analyses, confirm that the following items are present in the figure legend, table legend, main text, or Methods section.

n/a	Confirmed	
☐	☒	The exact sample size (*n*) for each experimental group/condition, given as a discrete number and unit of measurement
☐	☒	A statement on whether measurements were taken from distinct samples or whether the same sample was measured repeatedly
☐	☒	The statistical test(s) used AND whether they are one- or two-sided<br>*Only common tests should be described solely by name; describe more complex techniques in the Methods section.*
☐	☒	A description of all covariates tested
☐	☒	A description of any assumptions or corrections, such as tests of normality and adjustment for multiple comparisons
☐	☒	A full description of the statistical parameters including central tendency (e.g. means) or other basic estimates (e.g. regression coefficient) AND variation (e.g. standard deviation) or associated estimates of uncertainty (e.g. confidence intervals)
☐	☒	For null hypothesis testing, the test statistic (e.g. *F*, *t*, *r*) with confidence intervals, effect sizes, degrees of freedom and *P* value noted<br>*Give P values as exact values whenever suitable.*
☐	☒	For Bayesian analysis, information on the choice of priors and Markov chain Monte Carlo settings
☐	☒	For hierarchical and complex designs, identification of the appropriate level for tests and full reporting of outcomes
☐	☒	Estimates of effect sizes (e.g. Cohen's *d*, Pearson's *r*), indicating how they were calculated

Our web collection on statistics for biologists contains articles on many of the points above.

Software and code

Policy information about availability of computer code

Data collection	None
Data analysis	List of programs and softwares:<br>• R: version 4.1.1<br>• BWA-MEM: version 0.7.17 (https://sourceforge.net/projects/bio-bwa/)<br>• cgpCaVEMan: version 1.13.14/1.14.1/1.15.0/1.15.1 (https://github.com/cancerit/CaVEMan)<br>• cgpPindel: version 3.3.0/3.5.0 (https://github.com/cancerit/cgpPindel)<br>• ASCAT NGS: version 4.2.1/4.3.2/4.3.3/4.5.0 (https://github.com/cancerit/ascatNgs)<br>• cgpVAF: version 2.4.0 (https://github.com/cancerit/vafCorrect)<br>• Julia language: https://julialang.org/<br>• treemut (v1.1, https://github.com/NickWilliamsSanger/treemut)<br>• hdp (v0.1.5, https://github.com/nicolaroberts/hdp)<br>• GenomicRanges (v1.46.1), IRanges (v2.28.0), Rsamtools (v2.10.0), MASS (v7.3-55), stringr (v1.4.1), dplyr (v1.0.10), tidyr (v1.2.1), ape (v5.6-2), deconstructSigs (v1.8.0), ggplot2 (v3.4.0), MutationalPatterns (v3.4.1, 10.18129/B9.bioc.MutationalPatterns), gridExtra (v2.3), ggrepel (v0.9.2), RColorBrewer (v1.1-3), tibble (v3.1.8), dichromat (v2.0), seqinr (v4.2-16), phytools (v1.2-0), devtools (v2.4.5), lmerTest (v3.1-3), phangorn (v2.10.0), optparse (v1.7.3), parallel (v4.1.3)<br>Custom code made available (also stated in manuscript): https://github.com/mspencerchapman/Prolonged_persistence_of_DNA_lesions<br>No commercial software used.

For manuscripts utilizing custom algorithms or software that are central to the research but not yet described in published literature, software must be made available to editors and reviewers. We strongly encourage code deposition in a community repository (e.g. GitHub). See the Nature Portfolio guidelines for submitting code & software for further information.

Data

Policy information about availability of data

All manuscripts must include a data availability statement. This statement should provide the following information, where applicable:

- Accession codes, unique identifiers, or web links for publicly available datasets
- A description of any restrictions on data availability
- For clinical datasets or third party data, please ensure that the statement adheres to our policy

Sequence data that support the findings of this study have been deposited in the European Genome-Phenome Archive (https://www.ebi.ac.uk/ega/home) under the accession numbers relating to the original studies. These are listed below:
The longitudinal dynamics and natural history of clonal haematopoiesis (WGS Accession number EGAD00001007684)
Life histories of myeloproliferative neoplasms inferred from phylogenies (WGS Accession number EGAD00001007714)
Clonal dynamics of haematopoiesis across the human lifespan (WGS Accession number EGAD00001007851)
Convergent somatic mutations in metabolism genes in chronic liver disease (WGS Accession number EGAD00001006255)
Tobacco smoking and somatic mutations in human bronchial epithelium (WGS Accession number EGAD00001005193)
Lineage tracing of human development through somatic mutations (WGS Accession number EGAD00001006162)
Clonal dynamics after allogeneic haematopoietic cell transplantation using genome-wide somatic mutations (WGS accession number TBC)
The long-term effects of chemotherapy on normal blood cells (WGS accession number TBC)
All scripts and downstream data matrices required to reproduce the figures are available on github (https://github.com/mspencerchapman/Prolonged_persistence_of_DNA_lesions).
hg37 human reference genome has been used, as per all the original studies.

Field-specific reporting

Please select the one below that is the best fit for your research. If you are not sure, read the appropriate sections before making your selection.

☒ Life sciences ☐ Behavioural & social sciences ☐ Ecological, evolutionary & environmental sciences

For a reference copy of the document with all sections, see nature.com/documents/nr-reporting-summary-flat.pdf

Life sciences study design

All studies must disclose on these points even when the disclosure is negative.

Sample size	We included all available datasets that had (1) multiple phylogenies built from whole genome sequencing data that included large numbers of samples per phylogeny (>30), (2) for which there was some clonal structure after the initial period of development in at least some of the dataset, to allow detection of prolonged DNA lesions.
Data exclusions	Data exclusions were done as per the original studies, with analysis done on the filtered dataset.
Replication	To ensure the robustness of the findings, analysis was done in multiple datasets including distinct cohorts analysed at different times.
Randomization	This is not relevant to our study. Samples were included as per the original studies.
Blinding	Blinding was not relevant to our study. There was no test performed that required blinding.

Reporting for specific materials, systems and methods

We require information from authors about some types of materials, experimental systems and methods used in many studies. Here, indicate whether each material, system or method listed is relevant to your study. If you are not sure if a list item applies to your research, read the appropriate section before selecting a response.

Materials & experimental systems

n/a	Involved in the study	
☒	☐	Antibodies
☒	☐	Eukaryotic cell lines
☒	☐	Palaeontology and archaeology
☒	☐	Animals and other organisms
☐	☒	Human research participants
☒	☐	Clinical data
☒	☐	Dual use research of concern

Methods

n/a	Involved in the study	
☒	☐	ChIP-seq
☒	☐	Flow cytometry
☒	☐	MRI-based neuroimaging

Human research participants

Policy information about studies involving human research participants

Population characteristics	The dataset comprised 11,429 whole genomes from 89 individuals. Each phylogeny was generated from a single tissue type: haematopoietic stem and progenitor cells (HSPCs, n=39), bronchial epithelial cells (n=16) or liver parenchyma (n=48, from 34 individuals, due to separate phylogenies for 8 anatomical segments of the liver in 2 subjects). The HSPC phylogenies were from individuals that fell into five categories: foetal and cord blood (n=4), healthy adults (n=13), stem cell transplant donor/recipient pairs (n=10), patients with myeloproliferative neoplasms (n=10) and chemotherapy-exposed patients (n=2). Detailed metadata is available in Table S1.
Recruitment	Recruitment procedures were as outlined in the original studies (see references 9 - 16).
Ethics oversight	As per the original studies (see references 9 - 16).

Note that full information on the approval of the study protocol must also be provided in the manuscript.
